# Climate of Barbadoes—Phthisis

**Published:** 1829-01-01

**Authors:** 


### XXXI.

### CLIMATE OF BARBADOES—CONSUMPTION.

We have lately had an opportunity of seeing three or four individuals, who, having repaired to Madeira for the relief or arrest of incipient phthisis, voyaged thence to Barbadoes, where they sojourned during the cool months of December, January, and February, with great benefit to their complaints. They found themselves (so they thought) better at Barbadoes, than either at Madeira or on the ocean—chiefly on account of the greater facility of taking exercise in the tropical island, in consequence of its more level surface than that of Madeira. The following particulars of the climate of Barbadoes, condensed from a contribution of Dr. C. Thomas to Dr. Farre, may prove interesting to many of our readers.

The greatest barometrical variation, during a period of six years, did not exceed a quarter of an inch. The hygrometrical variations were not worth noticing. The thermometer seldom rises higher than 88° at noon, or falls lower than 70° in the coolest mornings. The island is situated in the 13th degree of north latitude, and 59th of west longitude. It is 21 miles long, by 14 in breadth. This island may be divided into the hilly and the flat districts.

"The low flat land occupies the northern, southern, and western parts of the island, and rises by precipitous broken acclivities running generally parallel to the coast in terraces of flat open country to the highest land, Mount Hallaby, situate near the center of the island. This progressive rise is only interrupted by one valley of any importance, crossing the island from the windward coast to Bridgetown, which lies to the south-west on the northern point of Carlisle Bay. Mount Hallaby is a rocky cliff, whose perpendicular altitude is upwards of 900 feet. From this point two steep precipitous ridges branch off to the north-east and south-east, including a country altogether different from the flat open scenery of the other district. The hills are numerous, lofty, conical and steep; the deep valleys intersecting them are covered with the most luxuriant vegetation; and the scenery is everywhere wild, irregular, and picturesque."

The substratum of the flat country is calcareous—the soil of the hills lies on the mineral substances belonging to the clay genus, and a variety of the ores of iron. There are no marshes or wet lands of any importance.

"The atmosphere is in general serene and clear, seldom cloudy; and the air is accordingly very healthy and dry: 'but what chiefly conduces to its purity is the regularity of the trade-winds, which seldom varying throughout the year further from the east than to the east-north-east, and consequently passing over a vast tract of water, must necessarily blow upon the island in cool refreshing gales. At the sun's first rising, they gently fan the air, and increase in strength in proportion to the active influence of the sun's heat, till towards evening, when the solar heat is abated, these refreshing gales, generally speaking, die away.'* The sea

* Hughes's Natural Hist. of Barbadoes.

breeze however sometimes blows by night as well as by day. On the other hand, there is no land breeze at Barbadoes, a circumstance easily accounted for by the want of any mountains of sufficient altitude to occasion it."

The rainy season begins in October. "It is then that the Heavens pour down cataracts." The hurricanes occur between the beginning of August and the latter end of October. Towards the end of November the north wind acquires force enough to clear the atmosphere, when a succession of serene and pleasant weather sets in, "spreading coolness and delight throughout the whole of this burning region." From the beginning of December, then, till the end of April, it is what may be called a tropical Winter, when, (to use the language of Bryan Edwards,) "to valetudinarians and persons advanced in life it is the climate of Paradise."

The foregoing information may be turned to advantage by medical men as well as by invalids. Patients affected with tubercles in the lungs, not yet advanced to the softened state—or those who have had hæmoptysis, but who have not actually purulent expectoration, might, with great advantage, embark for Barbadoes about the latter end of October, and sojourn in that island till the middle or end of April, when the voyage to Europe might be safely undertaken. This would probably be a less expensive, and a more salutary measure than a journey to Italy, and a residence during our Winter.

---

